# Copper laser patterning on a flexible substrate using a cost-effective 3D printer

**DOI:** 10.1038/s41598-022-25778-y

**Published:** 2022-12-07

**Authors:** Sajal Chakraborty, Ho-Yeol Park, Sung Il Ahn

**Affiliations:** Department of Chemistry Education, Graduate Department of Chemical Materials, Institute for Plastic Information and Energy Materials, Pusan National University, Busandaehakro 63-2, Busan, 46241 Republic of Korea

**Keywords:** Surface patterning, Electronic materials, Laser material processing

## Abstract

We studied the cost effective direct laser patterning of copper (Cu) on thin polyimide substrates (PI thickness: 12.5–50 µm) using a 405 nm laser module attached to an inexpensive 3D printer. The focal length of the laser was intentionally controlled to reduce defects on patterned Cu and surface damage of PI under predetermined process conditions. The appropriate focal length was examined at various focal distances. Focal distances of − 2.4 mm and 3 mm were found for the shorter focal length (SFL) and longer focal length (LFL), respectively, compared to the actual focal length. This resulted in clean Cu line patterns without line defects. Interestingly, the SFL case had a different Cu growth pattern to that of LFL, indicating that the small difference in the laser incident angle could affect Cu precursor sintering. Cu square patterns had a lower resistivity of 70 μΩ·cm for an LFL after three or four laser scans, while the SFL showed a resistivity below 48 μΩ·cm for a one-time laser scan. The residues of the Cu precursor on PI were easily removed with flowing water and normal surfactants. However, the resistivity of the patterns decreased after cleaning. Among the scan gaps, the Cu square pattern formed at a 70 μm scan gap had the lowest sheet resistance and the least change in resistance from around 4 to 4.4 Ω/ϒ after cleaning. This result implies that the adhesion of the patterned Cu could be improved if the coated Cu precursor was well sintered under the proper process conditions. For the application of this method to bioelectronics, including biosensors, LEDs were connected to the Cu patterns on PI attached to the arm skin and worked well, even when the substrate PI was bent during power connecting.

## Introduction

Small, portable devices on sensitive and flexible substrates require direct patterning processes instead of lithographic processes that require vacuum deposition, photoresists, and toxic chemical etching^1–7^. Thus, direct patterning processes have been researched extensively using metal nanoparticle inks, such as silver (Ag) and gold (Au)^8–10^. Instead of expensive noble metals, copper (Cu)-based composites attract attention due to their excellent thermal and electrical properties and cost-effectiveness^11–13^. However, due to their low oxidation potential energy (0.34 V) in comparison to the noble metals (Au, 1.52 V; Ag, 0.799 V), they have the disadvantage of easy oxidation under air^11^. Therefore, thermal sintering cannot be used to reduce the Cu precursor in an ambient environment. Recently, numerous attempts have been reported as an alternative thermal sintering method. One effective technique is protecting and stabilizing Cu-NP by applying a protective coating^14,15^ or using a thin noble metal in a core–shell structure^16,17^. The Cu precursor can also be sintered using high-power flash lamps, which can easily be integrated into massive production lines^18–21^. Since this method can heat the Cu precursor to a high temperature for full sintering within a few milliseconds, which can minimize the oxidation of Cu under air and impact polymer-based substrates, this photonic sintering is an attractive option for large-area printed electronics. Nevertheless, flash lamps emit strong, broad-spectrum light, which can cause partial deformation of polymer-based substrates. However, lowering the flash intensity to mitigate this risk will reduce the sinter quality. Moreover, this method is not a direct patterning method, and an additional patterning process is required before and after Cu sintering.

Another promising technique is direct laser sintering^22–27^. During focus, the focused beam energy is absorbed by the precursor and induces a localized, transient heating process that results in rapid sintering. Consequently, metal patterns that can be limited in resolution by the optical system and metal precursors can be achieved. Fast scanning can deliver high-resolution metal patterns that are a few micrometers wide. Furthermore, in ambient conditions, Cu oxidation is prevented if the sintering time is short enough. Un-sintered ink is easily removed by washing, which completes the patterning process. However, this process can damage polymer-based substrates due to the use of a focused laser with high energy density.

**Figure 1:**
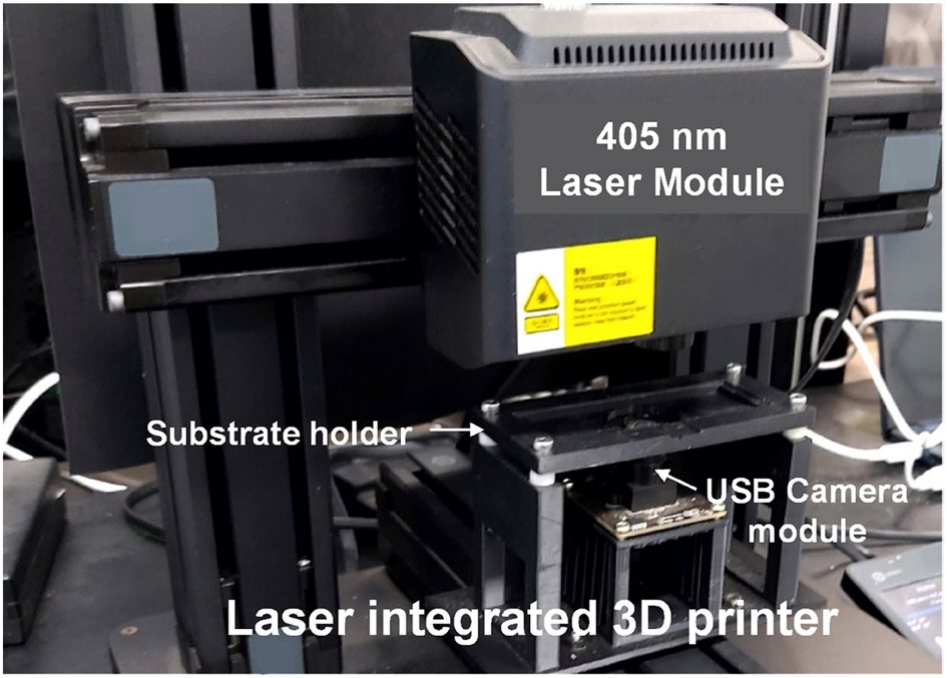
The laser-integrated 3D printer with a USB camera attached.

We tried to pattern Cu on polymer substrates with minimized surface damage using a metal–organic Cu precursor and an original laser module (405 nm diode, 500 mW) supplied by the printer manufacturer (Fig. 1). As products become lighter and smaller in size, many studies in the electronics field are underway to use polyimide (PI) as a lightweight and flexible polymer substrate to replace the current glass substrate^28–33^. This is because polyimide has many advantages, such as mechanical strength, chemical resistance, heat resistance, and thermal stability based on a rigid aromatic main chain^29^. Aromatic PIs have a yellowish or brownish color due to the imide ring and hence the charge transfer complex between the electron acceptor di-anhydride and the electron donor diamine present in the imide backbone^31–33^. Since the 405 nm laser used in this study can damage the surface of colored PI substrates more easily compared to transparent polymer substrates, we selected this colored PI film to test whether the surface damage can be minimized in the laser direct patterning process of Cu. When the laser was applied to form Cu patterns on flexible substrates, it caused severe damage to the substrate, even at a laser power below 10 mW, resulting in local burning or cleavage of the film. Therefore, we studied and developed a method to obtain Cu patterns using the proper process conditions to minimize substrate damage. Changing the 405 nm laser to one with a different wavelength that causes less damage was not considered. This is because among laser diodes with an output power of about 1 W, the 405 nm wavelength laser diode is the easiest to get and cheapest to use, satisfying our research purpose—the development of a cost effective and easy-to-use direct patterning machine and method. We also tried to develop process conditions to optimize the conductivity of patterned Cu while minimizing surface damage to the thin PI film.

## Results and discussion

Previous research indicates that Cu compounds are easily reduced at low temperatures when heat-treated under a nitrogen atmosphere. The decomposition chemistry and formation of metallic Cu of amine-coordinated Cu formate compounds is simplified by Eq. (1):^34,35^

1 $${\text{Cu}}\left( {{\text{HCOO}}} \right)_{{2}} \left( {{\text{RNH}}_{{2}} } \right)_{{2}} \to {\text{Cu}} + {\text{2CO}}_{{2}} + {\text{H}}_{{2}} + {\text{2RNH}}_{{2}}$$

**Figure 2:**
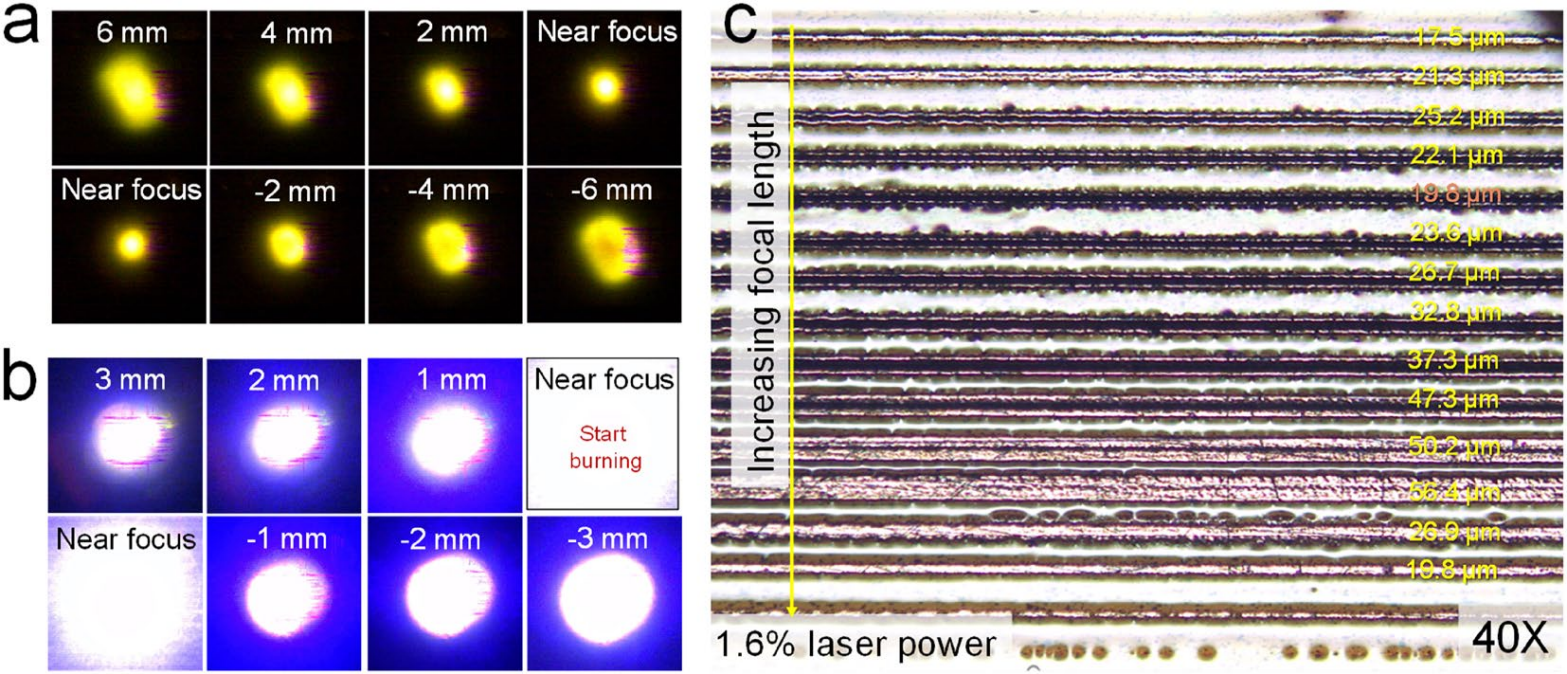
Focusing the laser beam: (**a**) Finding the laser focal length using a USB camera, (**b**) determining the focal length using a burning point on bare PI film at 1.6% pulse width modulation (PWM) input signal, and (**c**) determining the focal length through Cu patterns formed at various focal lengths using a G-code program (scan speed of 1 mm/s and 2% PWM).

Since sintering simultaneously accompanies the oxidation of Cu, a short heat treatment or nitrogen environment is required to prevent oxidation of the as-formed Cu. For laser direct patterning, the degree of sintering of the Cu precursor is related to several factors of the machine, such as the laser focal length, scan speed, and laser power. In addition, these factors influence each other. Laser power is the most important factor in controlling PI film damage. In fact, 1.6% of laser power (about 8 mW) at the actual focal length (AFL) can damage the PI (refer to the laser-focusing methods). In this study, by intentionally increasing or decreasing the focal length of the laser relative to the AFL, we tried to minimize defects in the patterned Cu and damage to the PI film. Therefore, finding the precise AFL of the laser first was necessary. Three different methods were introduced to achieve this. In the first method, a laser at 2% PWM input power was focused on a glass substrate covered with yellow PI and white polyethylene tape to find the smallest laser spot while controlling the z-axis (Fig. 2a) using a USB camera attached under the substrate. In the second method, we determined the focal length using a burning point on bare PI film at 2% input power while gradually decreasing the z-axis (Fig. 2b). From the above two methods, a rough focal length was found. However, for precise focusing, the third method involved programming a G-code file based on the focal length from the above two methods. Subsequently, we conducted line patterning of Cu at different heights around the rough focal length and determined the AFL of the laser. Figure 2c is a microscope image of Cu lines formed at different focal lengths and a constant input of 1.6%. Among the Cu lines, the narrowest line width of Cu is found near the AFL of the laser, and we observed various defects in the Cu patterns and on the PI film. Similar to previous reports^21–23^, the most common defects are line defects, which are empty or perforated areas in the middle of patterned Cu lines. We found that defective patterns appear differently along with up and down directions from the AFL. The longer focal length (LFL) region exhibits more severe line defects than the shorter focal length (SFL) region. In addition, Cu lines gradually increase in width in both directions as the distance from the AFL increases. Some of the defects are accompanied by surface damage to the PI film. The strong energy of a well-focused laser beam is a probable cause, even at the low laser power of 1.6% (about 8 mW). However, a proper focal length different from the AFL exists, and it gives clean Cu patterns at a constant laser power (Fig. 2c).

**Figure 3:**
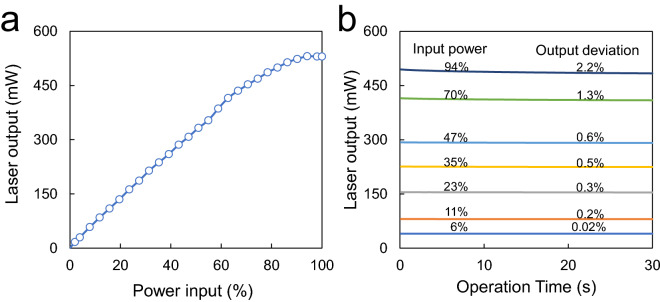
Characteristics of laser output power against input signals: (**a**) Laser output against PWM input ratios and (**b**) laser output fluctuations at various PWM ratios in the “on” state for 30 s.

Laser power stability is an important factor in improving the quality of patterns. We tested laser output depending on input signals controlled by a PWM method. The output power of a laser module linearly increased up to around 70% PWM signal and then slightly decreased contrary to the expectation from the input, probably due to the cooling ability of the laser module (Fig. 3a). Figure 3b shows that the deviation of laser output increases with increased input power signal (measured for 30 s after turning the laser on). Since the input power used in this study is 38% (around 260 mW) of the maximum power, determined by a later experiment, the power deviation of the laser is expected to be much smaller than 0.6% since the “turn on” time of the laser is less than a few seconds based on a scan speed of 4 mm/s in this experiment.

**Figure 4:**
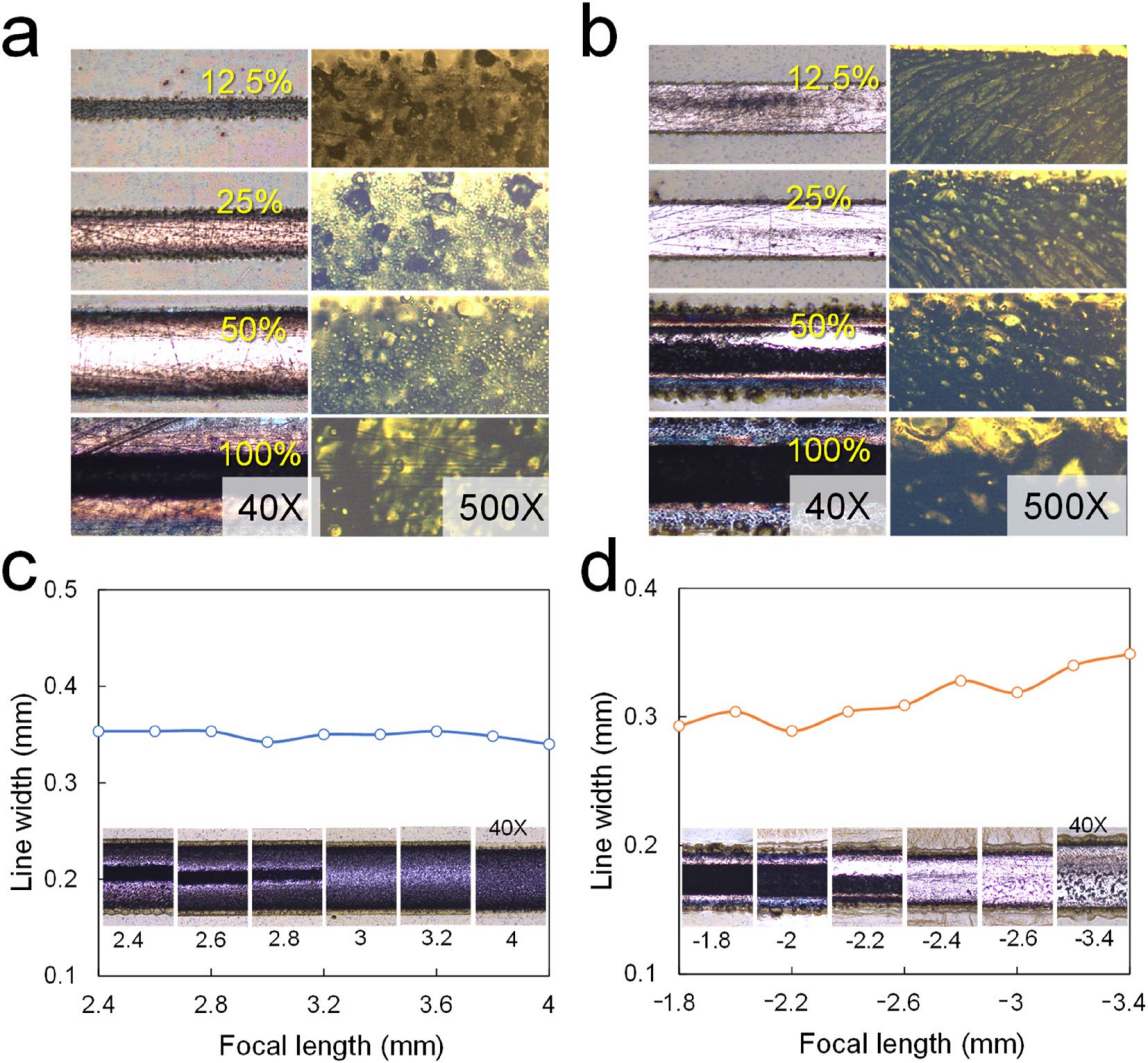
Microscope images of patterned Cu as a function of input PWM signal ratios from 12.5 to 100%: (**a**) Cu lines formed at LFL and 4 mm/s scan speed, (**b**) at SFL and 4 mm/s scan speed. Microscope images and linewidth of Cu patterns formed at various SFLs and LFLs to find the proper focal distance to minimize defects: (**c**) Focal distance (LFL) from 2.4 to 4 mm, (**d**) focal distance (SFL) from − 1.8 to − 3.4 mm based on the RFL. Note that the Cu patterns were obtained at 38% PWM input and 4 mm/s scan speed.

The shape and defects of the Cu patterns were investigated while increasing the laser power at a constant scanning speed and a fixed focal length (LFL or SFL). For the LFL case (3 mm longer than the AFL) in Fig. 4a, the linewidth and grain size of Cu increases with increasing laser power, and line defects reappear in the Cu pattern at above 50% input power. For the SFL case (2.4 mm shorter than the AFL) in Fig. 4b, similar results to the LFL case are observed, except that laser marks appear on the Cu pattern. Interestingly, the linewidth increase rate of the SFL with respect to the laser power in Fig. 4c appears smaller than that of the LFL because the angle and diameter of the incident laser beam are different. Based on these results, including other preliminary tests, a 38% PWM power input was selected for the Cu direct laser patterning.

To find the appropriate SFL and LFL, the copper pattern was investigated at various focal distances using a G-code file. Figure 4c–d show the line width and microscope images of Cu patterns in the SFL and LFL regions obtained at a 38% input power and 4 mm/s scan speed. The SFL shows bigger defect lines on Cu patterns than the LFL. Much more long or short than the AFL, we can find aggregates of carbon residues and Cu. Figure 4 shows that both cases produce Cu patterns without defects at a certain focal distance. Based on this result, we fixed the focal lengths at around − 2.4 mm for SFL and 3 mm for LFL and conducted further experiments.

**Figure 5:**
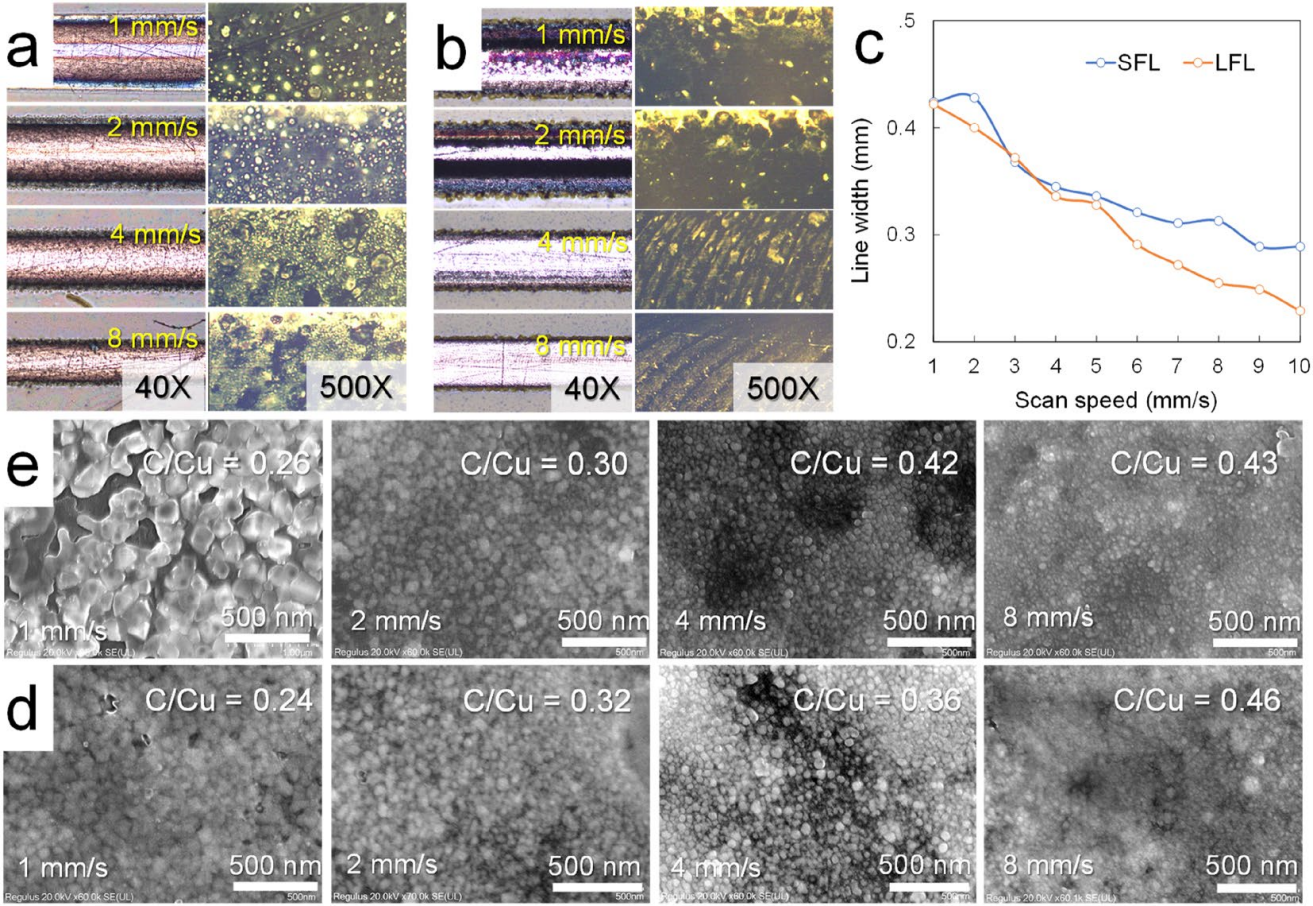
Microscope and scanning electron microscope (SEM) images of patterned Cu as a function of laser scan speed from 1 to 8 mm/s at 38% PWM input: (**a**) Cu lines formed at LFL, (**b**) Cu lines formed at SFL, (**c**) line widths of Cu patterns against the scan speed, (**d**) SEM images of LFL, and (**e**) SEM images of SFL against the scan speed. The insets in (**a**) and (**b**) are enlarged images of each Cu pattern.

The laser irradiation time is directly related to the scan speed (or velocity) of the laser module attached to the 3D printer. We examined Cu patterns while increasing the scan speed at 38% power input and a predetermined focal distance. In Fig. 5a, the LFL case clearly shows an increase in the particle size of Cu at a low scan speed and a decrease in the linewidth with increasing scan speed. The SFL case in Fig. 5b has a similarly decreased linewidth but a lower change rate of linewidth than the LFL case (Fig. 5c). Both cases had defects at a scan speed below 2 mm/s due to the prolonged laser dwelling time, while from 4 mm/s, they showed clean Cu patterns. Interestingly, the SFL has a different Cu line pattern to that of the LFL, indicating that the small difference in the incident angle of the laser can affect Cu sintering, similar to the previous power test results. The SEM images of SFL and LFL in Fig. 5d–e match the microscope images, indicating that Cu particle size increases as the scan speed decreases. In addition, energy-dispersive X-ray spectroscopy (EDS) analysis shows that the C content in the Cu pattern increases with an increasing scan rate. Based on these results, the scan speed was determined to be 4 mm/s.

**Figure 6:**
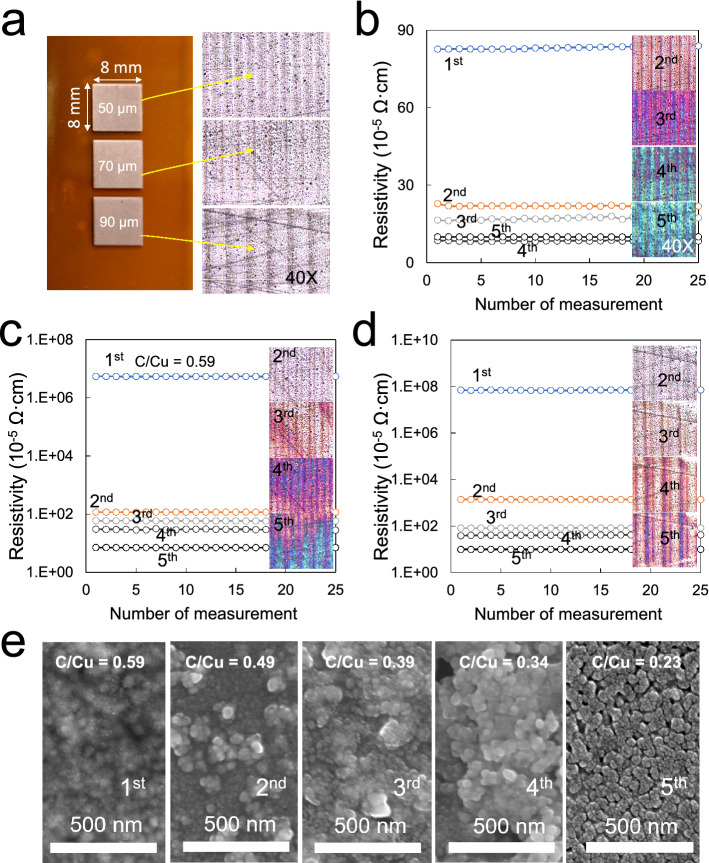
The resistivity of Cu square patterns formed at different scan gaps (50, 70, and 90 μm) and LFL: (**a**) Camera images of Cu square (8 × 8 mm^2^) patterns on PI and their microscope images, (**b**) resistivity curves of the 50 μm scan gap, (**c**) 70 μm scan gap, and (**d**) 90 μm scan gap against several laser scans, (**e**) SEM images with C/Cu ratios of the 70 µm sample versus several laser scans.

After finding the appropriate conditions of the laser power, scan speed, and focal length, the resistivity of Cu patterns was measured from 8 × 8 mm^2^ square Cu patterns with different laser scan gaps (50, 70, and 90 μm). Figure 6a shows the camera images of Cu square patterns with the strip lines of carbon residues formed during sintering. A one-time laser scan at the LFL shows around 830 μΩ·cm at 50 μm, 5.4 Ω·cm at 70 μm, and 4.9 Ω·cm at 100 μm scan gaps. To fix this high resistivity and further examine the laser treatment effect, the Cu patterns were laser treated again. Further laser scans gradually decreased the resistivity of the Cu pattern, which dropped to 70 μΩ·cm (Fig. 6b). For the 70 and 90 μm line scan gaps, the resistivity drops sharply to about 70 μΩ·cm (Fig. 6c–d). The SEM images of the 70 μm sample in Fig. 6e show that the Cu particle size increases if the laser scan is extended. According to the EDX analysis of this sample, the C/Cu ratio also decreased with the number of laser scans.

**Figure 7:**
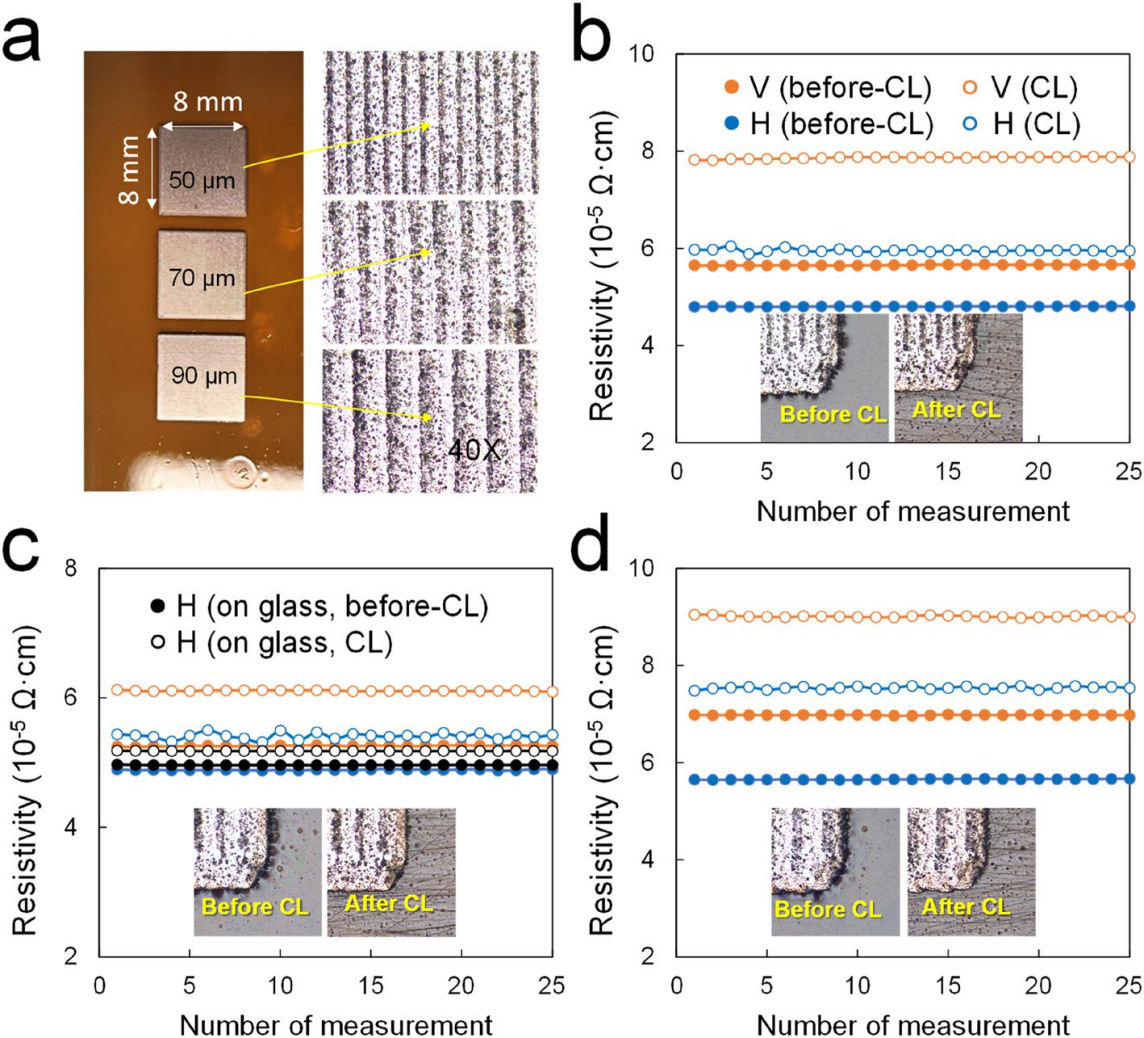
The resistivity of Cu square patterns formed at different scan gaps (50, 70, and 90 μm) and SFL: (**a**) Camera images of Cu square (8 × 8 mm^2^) patterns on PI and their microscope images, (**b**) resistivity curves of the 50 μm scan gap, (**c**) 70 μm scan gap, and (**d**) 90 μm scan gap. Note that the resistivity graph includes a comparison of the resistivity in the vertical/horizontal direction with respect to the laser scan direction and before/after cleaning for each case.

Figure 7a shows camera and microscope images of Cu square patterns formed at the SFL having carbon residue strip lines similar to that of the Cu patterns formed at the LFL. The Cu patterns have a completely different resistivity from that of LFL. Despite the scan gaps, all the patterns, after a one-time laser scan, have a much lower resistivity (below 60 μΩ·cm in Fig. 7b–d) than that of LFL. Surprisingly, this resistivity is comparable to that of the Cu square patterns formed on glass substrates obtained at 100% input power (Fig. 7c). One important aspect of using this patterning method is in removing residues, which are unreacted Cu precursors, from the PI substrate with minimal Cu pattern damage. Fortunately, the Cu precursor is removed well with flowing water and normal surfactants. The insets in Fig. 7b–d show the microscope images taken before and after cleaning the Cu patterns. In fact, the Cu precursor film has a bluish color, but it is hardly visible to the human eye or the microscope. However, compared to the as-formed Cu patterns with black residue on their edges, the cleaned patterns showed few residues. We measured two sheet resistances, vertical and horizontal, depending on the laser scan direction and found that the resistance of the sample increased slightly after washing. The resistivity changes in the horizontal direction were 0.8 Ω/ϒ at 50 μm, 0.4 Ω/ϒ at 70 μm, and 1.3 Ω/ϒ at 90 μm scan gaps. The resistivity changes in the vertical direction were 1.6 Ω/ϒ at 50 μm, 0.6 Ω/ϒ at 70 μm, and 1.4 Ω/ϒ at 90 μm scan gaps. Interestingly, among the scan gaps, the Cu pattern at 70 μm has the lowest resistivity and the least change in resistivity after washing. This result indicates that the adhesion of the patterned Cu can be improved if the Cu precursor is well sintered under suitable process conditions.

**Figure 8:**
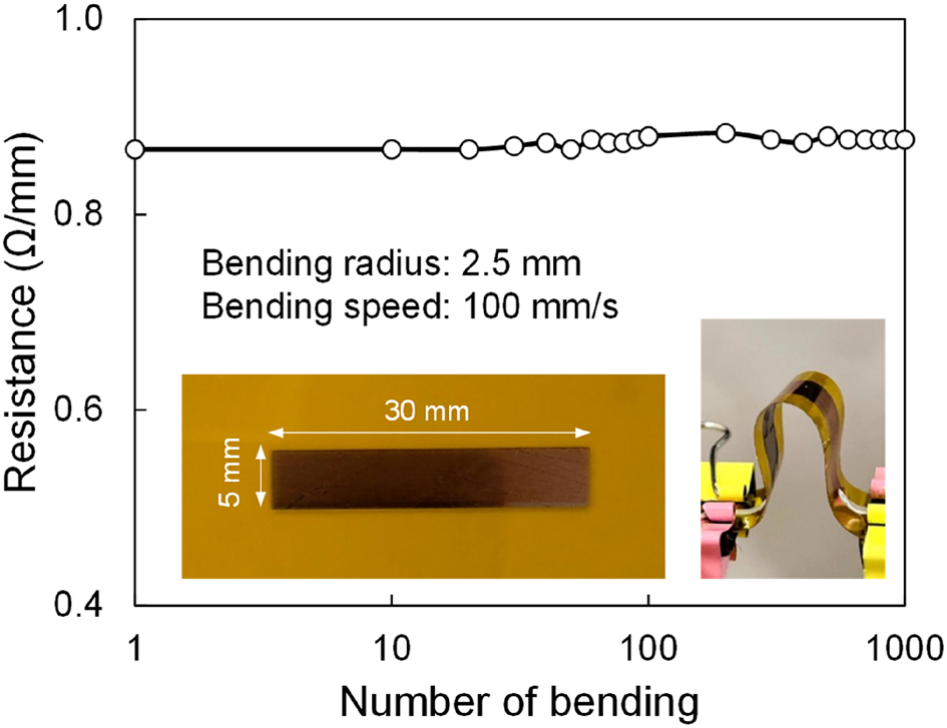
Resistance changes against several bending of the laser-sintered Cu. The bending test was performed at a bending speed of 100 mm/s and bending radius of 2.5 mm.

A square pattern of Cu with dimension of 5 × 30 mm^2^ on 25 μm PI was prepared, and bending tests were performed using a homemade bending machine. After 1000 bending (2.5 mm bending radius), the resistance of the sample changed from 0.87 to ~ 0.88 Ω/mm as shown in Fig. 8.

**Figure 9:**
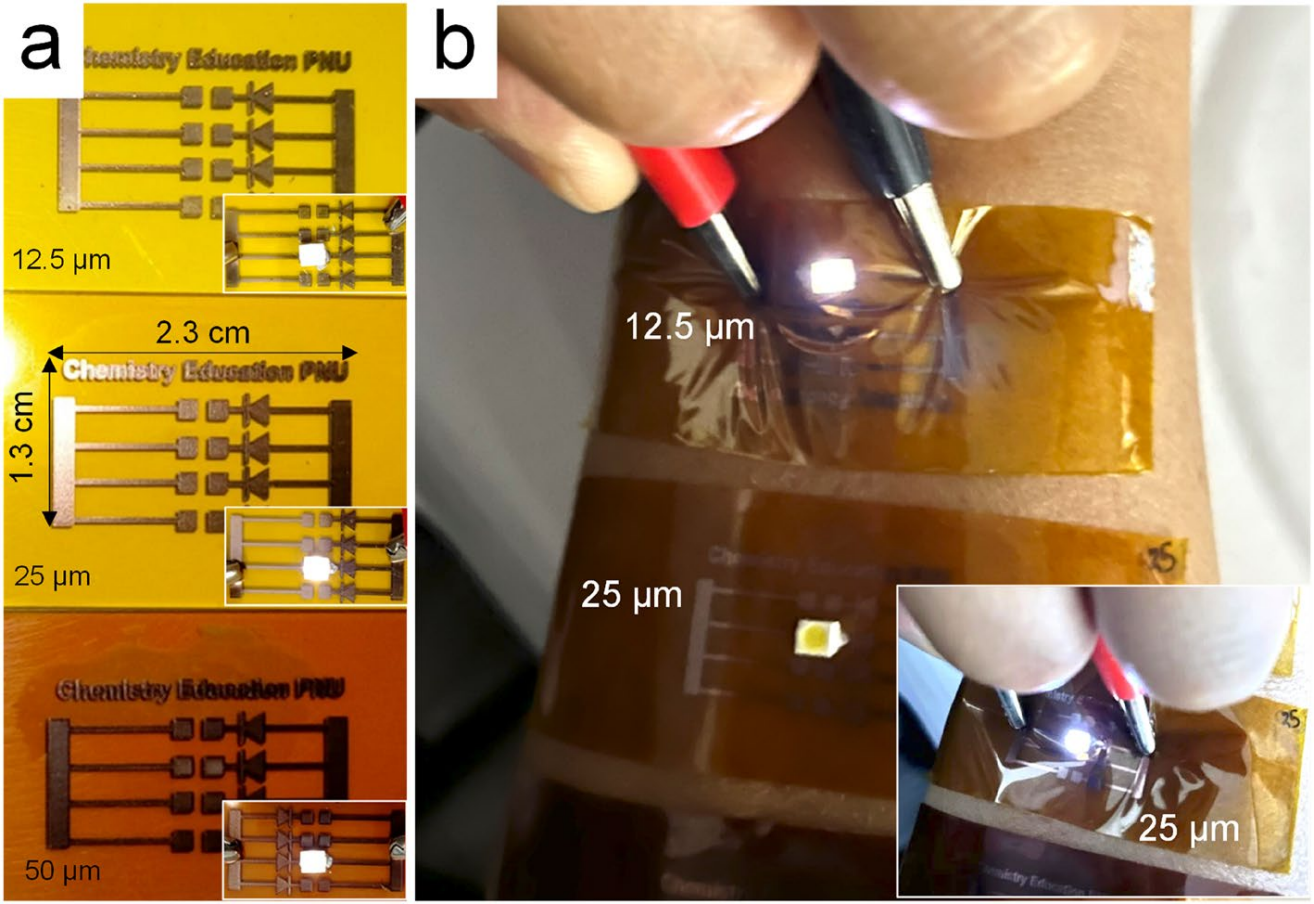
Various Cu patterns designed for testing electrical connections with LEDs: (**a**) Cu patterns formed on PI films with different thicknesses (12.5, 25, and 50 μm) attached to a glass plate and (**b**) LEDs operating on the Cu patterns of PI films attached to the arm skin.

Various patterns, including characters, were designed, and the Cu laser direct patterning was performed according to the predetermined process conditions. The Cu precursor was coated on different PI films with thicknesses of 12.5, 25, and 50 μm and sintered with the laser. Figure 9a shows well-defined Cu patterns in all PI films despite the thickness. To demonstrate that the Cu pattern can be used as a flexible electrode, a small LED was attached to the pattern and operated. The insets in Fig. 9a indicate that the LED works properly. One potential application of this electrode could be in small bioelectronics devices containing biosensors. Thus, after attaching PI with Cu patterns and LED to the arm skin, the same test was performed. The results confirmed that it operated well, even when the substrate PI was bent during power connecting (Fig. 9b).

## Conclusions

For the cost effective direct laser patterning of Cu on a flexible substrate of PI, we examined a cheap 3D printer (below USD $1000) with a laser module. Three methods were introduced to find the focal length of the laser: beam spot analysis using a USB camera, locating a burning point against the z-axis height, and utilizing Cu patterns formed at a different z-axis height using a G-code file. Laser power fluctuation was measured for various PWM signals and was below 0.6% deviation for 30 s at 38% PWM input. The appropriate focal length was investigated at various focal distances, and a focal distance resulting in clean Cu line patterns without line defects for the SFL and LFL was found. Based on this result, we fixed the focal lengths at around − 2.4 mm for the SFL and 3 mm for the LFL. From the scan speed test relative to the irradiation time of the laser, we found that prolonged dwelling of the laser resulted in an increased Cu particle size but defects in the Cu patterns. Interestingly, the SFL had a different Cu growth pattern to that of the LFL, indicating that the small difference in the laser incident angle could affect the sintering of Cu precursors. The Cu patterns obtained at the predetermined conditions had a minimum resistivity of 70 μΩ·cm for LFL after several laser scans, while the SFL showed a resistivity below 48 μΩ·cm for a one-time laser scan. This was a comparable value to that of the Cu square patterns on glass substrates formed at a 100% PWM signal. The unreacted Cu precursor on PI was removed well with flowing water and normal surfactants. However, when compared to the Cu patterns before cleaning, the resistivity of the pattern was found to have decreased. Interestingly, among the scan gaps, the Cu pattern formed at a 70 μm scan gap had the lowest resistivity and the least change in resistivity after washing. This implies that the adhesion of the patterned Cu could be improved if the coated Cu precursor was well sintered under the proper process conditions. One expected application of this method will be in fabricating bioelectronics, including biosensors. For this, the operation of the LED connected to the Cu patterns on PI attached to the arm skin was demonstrated, and the LED worked well, even when the substrate PI was bent during power connecting.

### Methods

A Cu MOD solution was fabricated in the following order with reference to a previous report.^30,31^ First, methoxyethanol (60 ml, Daejung Chemicals & Metals Co., Ltd) was mixed with monoethanolamine (8 ml, Daejung Chemicals & Metals Co., Ltd). Second, 4 g of Cu (II) formate tetrahydrate (Alfa Aesar) was dissolved in the solution for 10 min. To remove water and coordinate the Cu with the amine, this mixture was heat-treated at 140 °C for 35 min while stirring and subsequently cooled. The final volume of the MOD solution was around 21 and 23 ml. By adding distilled methoxyethanol to the solution, we obtained 25 ml of Cu precursor MOD solution. After filtering using a syringe filter (CHMLAB PVDF Syringe Filters, 0.45 μm), we spin-coated the solution on PI films (12.5, 25, and 50 μm in thickness, ISOFLEX PIT-S1206HS-50G-FL50, PIT-S2505HS-100G-FL50, and PIT-S5020HS-250G-FL50, respectively) attached on a sliding glass at 1200 rpm for 10 s and dried it on a hot plate at 130 °C for 90 s. For laser direct patterning, we used a commercial 3D printer (Mooz2, DOBOT) with a laser head installed; for simplicity, we used the original laser source of 405 nm provided by the manufacturer. The laser power was measured using a power meter (PowerMax-USB, COHERENT) for various input signals generated by the PWM method. To determine focal length and examine the patterning process, a USB camera was attached to a holder that had been 3D printed (Fig. 1) and positioned just below the substrate. Cu patterns on PI were produced at varying focal lengths, laser powers, printing speeds, and other factors using G-code, and then we determined the process conditions. The fine structure of Cu in the patterns was analyzed with a SEM (Hitachi Regulus 8100, JP) and EDS using an Oxford Ultim Max 40 (Oxford Instruments, UK), KLA-Tencor. Sheet resistances of the patterns were obtained using a four-point probe (OSSILA) method.

## Data Availability

The datasets used or analysed during the current study are available from the corresponding author on reasonable request.
